# Feasibility of 3 Tesla MRI for the assessment of mid-palatal suture maturation: a retrospective pilot study

**DOI:** 10.1007/s10266-024-00950-0

**Published:** 2024-05-17

**Authors:** Ines Willershausen, Markus Kopp, Michael Scholz, Armin Ströbel, Corinna Lesley Seidel, Friedrich Paulsen, Michael Uder, Lina Gölz, Matthias Stefan May

**Affiliations:** 1 Department of Orthodontics and Orofacial Orthopedics, Friedrich-Alexander-University Erlangen-Nuremberg, Erlangen, Germany; 2 Institute of Radiology, Friedrich-Alexander-University Erlangen-Nuremberg, Erlangen, Germany; 3 Institute of Functional and Clinical Anatomy, Friedrich-Alexander-University Erlangen-Nuremberg, Erlangen, Germany; 4 Center for Clinical Studies (CCS), Medical Faculty, Friedrich-Alexander University Erlangen-Nuremberg, University Hospital Erlangen, Erlangen, Germany

**Keywords:** Mid-palatal suture, MRI, Radiology, Orthodontics, Anatomy

## Abstract

The maxilla occupies a key position in dentofacial orthopaedics, since its transversal development can be directly influenced by orthodontic therapy. The maturation stages of the mid-palatal suture, which are obtained from cone-beam computed tomography images (CBCT), present an addition to clinical decision-making in transversal discrepancies of the upper jaw. In an endeavour to reduce ionizing radiation in adolescents and young adults, who are particularly susceptible to long term stochastic irradiation effects, we investigated the feasibility of 3 Tesla (3T) MRI in detecting the maturation stages of the mid-palatal suture. A collective of 30 patients aged 24–93 years with routine neck MRI at 3T, underwent an additional three-dimensional isotropic T1 weighted study sequence of the midface. Image evaluation was performed on axial, multi-planar formatted reconstructions of the dataset aligned to the midline axis of the palate, and curved reconstructions aligned to the concavity of the palate. Inverted images helped to achieve an image impression similar to the well-known CBCT appearance. All datasets were reviewed by three readers and mid-palatal maturation was scored twice according to Angelieri et al. Intra- and inter-rater agreement were evaluated to measure the robustness of the images for clinical evaluation. 3T MRI deemed reliable for the assessment of mid-palatal suture maturation and hence for the appraisal of the hard palate and its adjacent sutures. The data of this pilot study display the feasibility of non-ionizing cross-sectional MRI for the determination of sutural maturation stages. These findings underline the potential of MRI for orthodontic treatment planning, further contributing to the avoidance of unnecessary radiation doses.

## Introduction

**Fig. 1:**
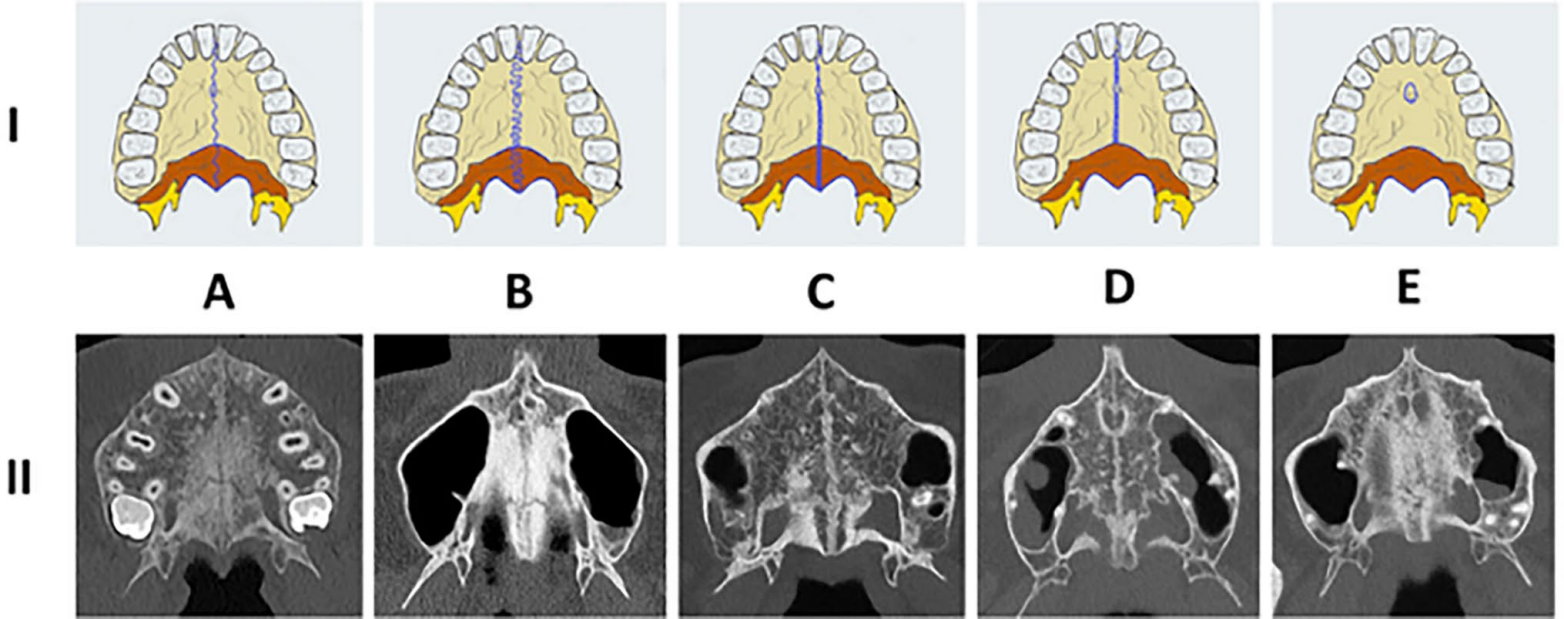
**I** displays a schematic drawing, (adapted from Angeleri et al. [17]) and **II** displays the corresponding cross-sectional images of the five maturation stages in CT. In **I,** the maxillary is colored in yellow while the palatine bones are depicted in orange and the suture is colored in blue, the sphenoid bone is colored in dark yellow. In the first 3 stages (A–C), the suture is visible in both the maxillary and palatal bones, while in stage (D), the suture in the palatal bone has already fused and in stage E complete fusion has taken place in both bones

The upper jaw is of the utmost importance for orthodontics and orofacial orthopaedics, since transversal and sagittal maxillary growth development can be actively influenced [1, 2]. Transversal palatal expansion is particularly intriguing, since this simultaneously widens the nasal floor [3]. As a consequence, it enhances nasal breathing and has been described to improve sleep apnoea in children and overall quality of life [4–7]. Rapid maxillary expansion is, therefore, considered to be an orthodontic procedure with a high impact on general health [8]. In children, who are treated before the peak of growth velocity, this intervention is efficient and predictable [9]. However, with increasing age, it becomes increasingly difficult to predict the success rate of transverse expansion as there is no linear correlation between patient age and suture fusion [10]. Starting in late adolescence, maxillary resistance becomes increasingly pronounced, so that a transversal deficiency can only be corrected with an additional subtotal Le Fort I osteotomy [11, 12]. Since the cut-off value for surgery is still very much empirical, sutural maturation stages have been classified histologically and radiologically to increase the predictability of rapid maxillary expansion in late adolescence [13–16]. Cross-sectional CBCT imaging has been described as a non-invasive method for the assessment of mid-palatal suture maturation in clinical routine [17]. Angelieri et al. were the first to use axial CBCT images in 2013 to describe 5 different stages of maturation, ranging from A, an open suture, radiologically depicted as a line of high density with little or no interdigitation (Fig. 1A–E) to E, a completely fused suture (Fig. 1E) [13, 17, 18]. The authors suggest that patients with stages D and E (Fig. 1D, E) as the most obliterated ones, will most likely experience inadequate success of treatment and iatrogenic side effects ranging from buccal tipping, recessions to gingival ulcers and will, therefore, benefit most from additional surgery [10, 17, 19, 20]. Although Angelieri et al. have introduced a promising approach towards individualised orthodontic treatment, a cone-beam computed tomography (CBCT) or computed tomography (CT) has to be carried out to obtain their index. According to the linear-non-threshold-model (LTN), ionizing radiation is always linked to an elevated stochastic cancer risk and children, who represent the phenotypical orthodontic patient collective, have been described to be particularly susceptible to irradiation side effects [21–25]. In recent years, MRI as a non-ionizing methodology to obtain cross-sectional images, has found increasing application in dental medicine and orthodontics [26–29]. This is mainly due to continuously increasing spatial resolution and image contrast as well as decreasing scan duration [30, 31]. MRI scanners are available at different B_0_-field strengths (magnetic flux density), ranging from 0.55 to 7 Tesla (T = kg⋅s^−2^⋅A^−1^) for clinical application. The feasibility of 1.5 T Black Bone MRI for the detection of mid-palatal suture maturation was first described by Rossi et al. in a preliminary study investigating one patient only [32]. Black bone MRI was considered to have clinical potential for assessment of the hard palate and adjacent sutures [32]. The aim of this retrospective study was to assess the feasibility of 3 T MRI in diagnosing the maturation stages of the mid-palatal suture investigating a larger sample size.

## Materials and methods

**Fig. 2:**
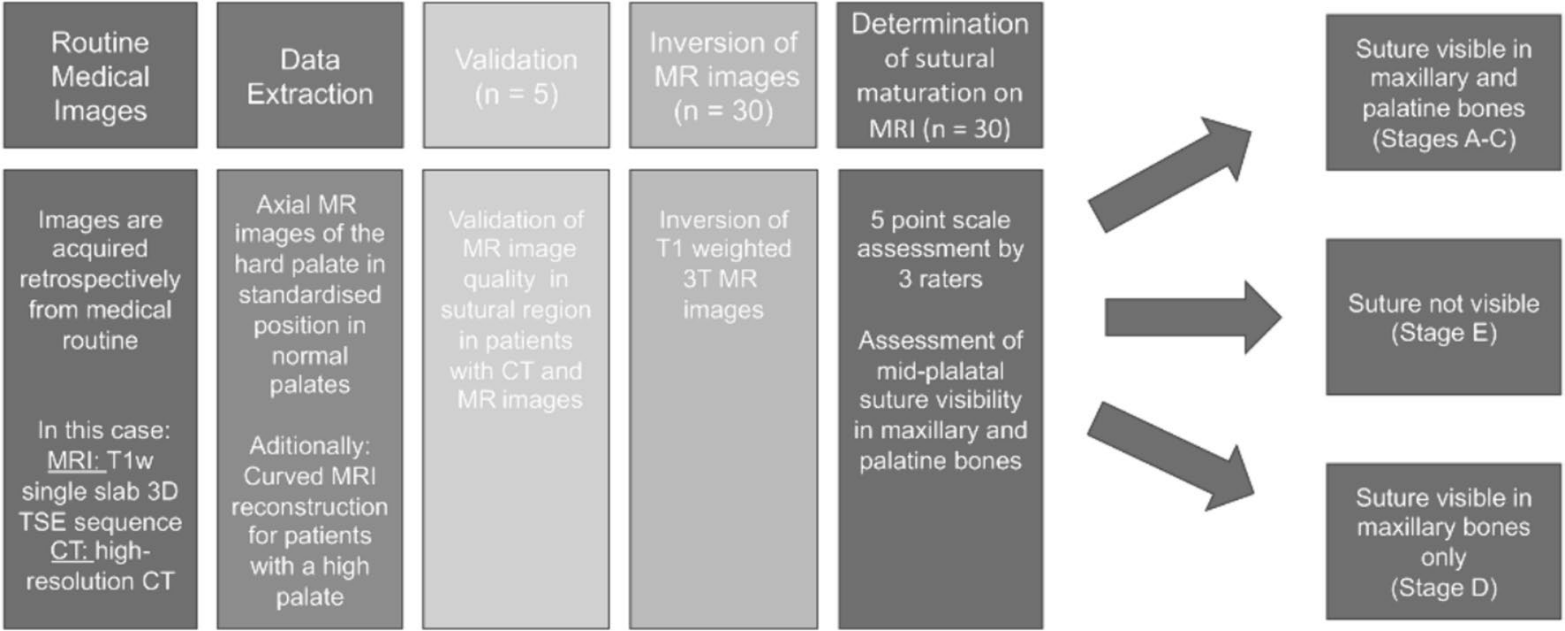
Displays the post-processing pipeline for validating sutural maturation status in T1w single slab 3D TSE sequence

**Fig. 3:**
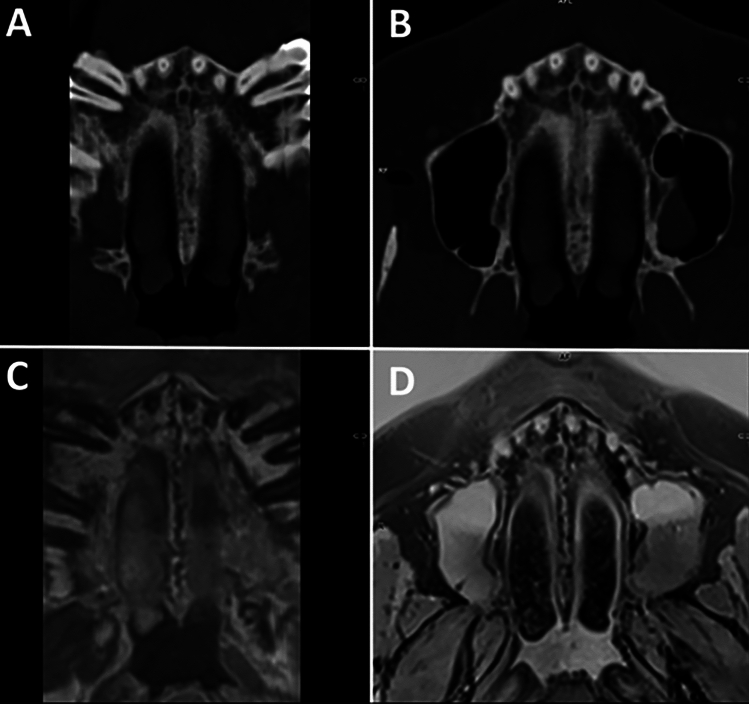
Displays the mid-palatal suture with maturation stage B. **A,** **B** Display the suture in axial CT, **C** in a T1w 3 T MRI and D shows an inverted image of the original MRI

**Fig. 4:**
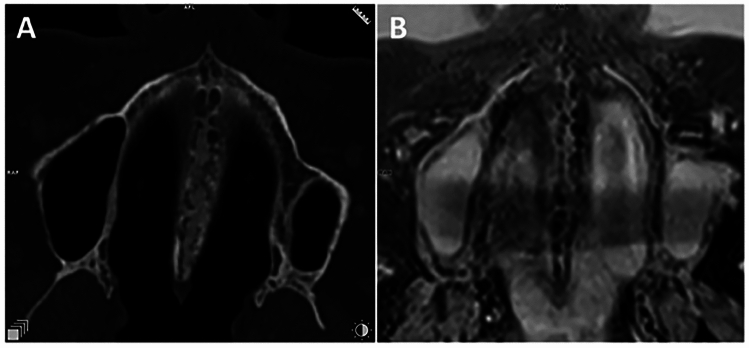
Displays the mid-palatal suture with maturation stage C. **A** Displays the suture in axial CT, **B** as an inverted image of the MRI

**Fig. 5:**
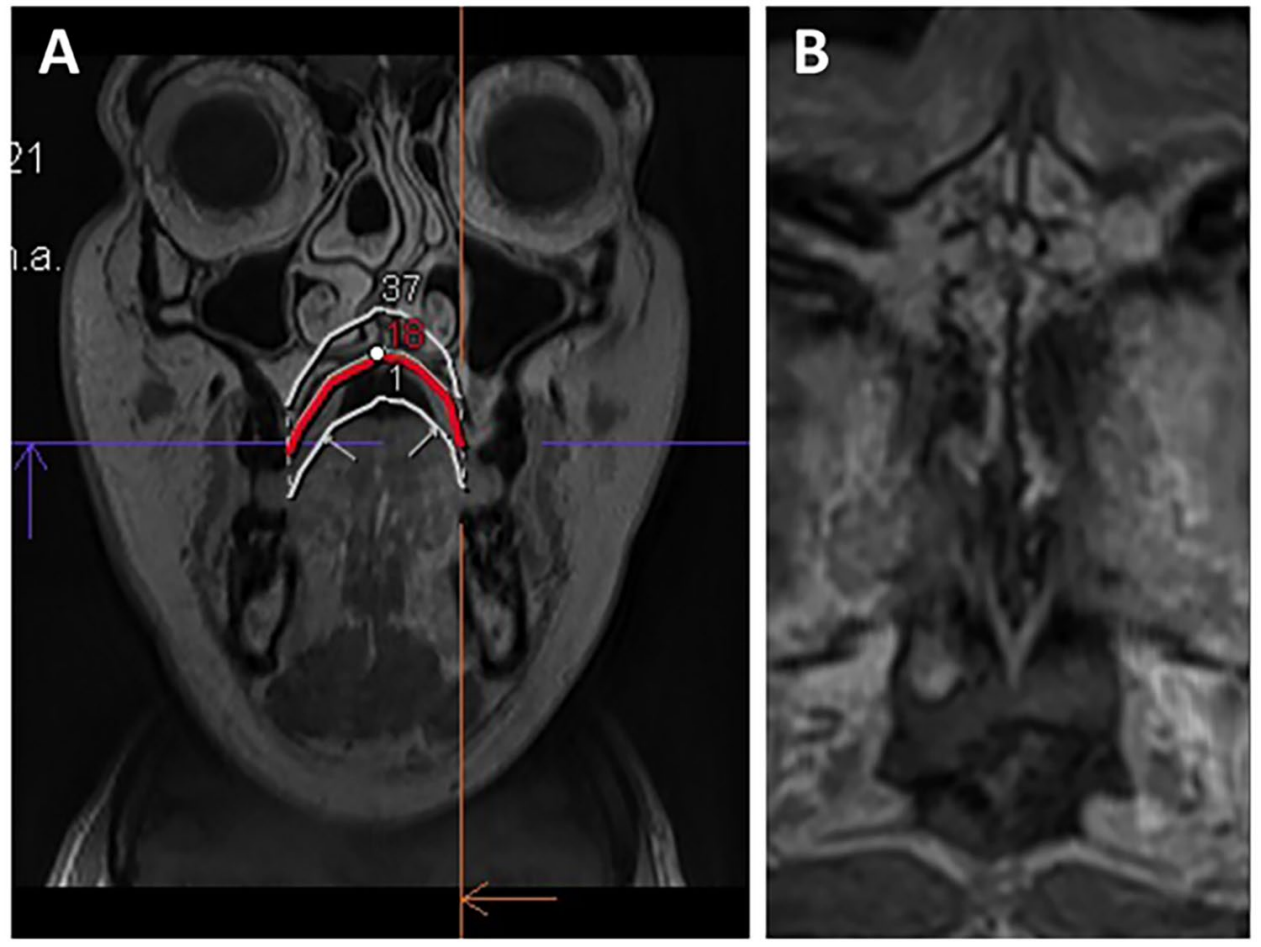
**A** Displays the curved MPR reconstruction of the mid-palatal suture in one plane **B**. This technique is particularly helpful in patients with a high or very thick palate, who would normally need 2 or even more axial images to obtain the index

After approval of the institutional review board (IRB Number: 22–338-Br), we retrospectively selected patients from our archives, who had received a diagnostic neck MRI and an additional study sequence of the midface, in a time period from March 2021 to July 2022. Patients were only enclosed if they had a full dentition, with a maximum of one singular tooth missing per jaw. Subjects with facial skull asymmetries, previous trauma or surgery of the midface, craniofacial malformations, displaced teeth in the palatal region and metabolic bone disorders were excluded. After screening the records of 50 applicable cases, a total of 30 patients met the inclusion criteria for further analysis. This sample size is in line with the rules of Koo et al. who consider a heterogeneous sample size of 30 people with 3 readers involved to be valid for a reliability study [33]. All of the patients had received a three-dimensional isotropic T1 weighted sequence, allowing for 3D reconstruction: T1w single slab 3D TSE sequence with slab selective, variable excitation pulse ((SPACE, Siemens Healthcare GmbH, Erlangen, Germany), TE 27 ms, TR 700 ms, flip angle mode: T1Var (Tissue T1 940/Tissue T2 100/Organ Exam Standard), field of view 250 × 250 mm, matrix 320 × 320)). The reconstructed voxel size was 0.4 × 0.4 × 0.8 mm. This sequence enables both, a good visualisation of the bony marrow structures and an individual 3D angulation to the hard palate, suitable for the detection of the five mid-palatal maturation stages according to Angelieri et al. [13, 17, 18]. In addition to the underlying MRI, a total of 5 patients had additionally received a high resolution CT (Siemens SOMATOM X.ceed; slice thickness 0.5 mm; tube voltage 100 kV) on account of a medical justifying indication. The images of these patients were employed to validate MR image quality of the sutural region as opposed to the CT images as the gold standard (Figs. 2, 3, 4). All of the MR images were subsequently inverted to yield a closer resemblance to the image impression on CBCT (Figs. 3, 4). The Angelieri maturation stages are scored on a five-point scale (stages A–E) (Figs. 1, 2) [17]. In stages A–C, the suture is visible in both the maxillary and palatine bones. Stage A is described as straight high-density line with no or little interdigitation (Fig. 1A) [17]. Stage B is defined as a scalloped high-density line with a phenotypically irregular shape (Fig. 1B) while in stage C, there are two parallel, scalloped, high-density lines close to each other (Fig. 1C) [17]. In stage D, sutural fusion has commenced in the palatine bone (Fig. 1D) and in stage E, the suture is totally fused (Fig. 1E) [17]. For the assessment of the above-mentioned indices, we reconstructed dedicated axial MR images, strictly abiding by the respective sagittal and coronal head orientation as proposed by Angelieri [17]. In addition, the 3D data sets could be adapted to the midline axis of the palate, using a standard multiplanar reconstruction (MPR) viewer. Furthermore, in patients with a particularly high or thick plate, a curved reconstruction was performed, aligning the plane with the concave shape of the palate in coronal view. This allows the comprehensive assessment of the suture in one single plane, even in cases of complex anatomy (Fig. 5). In a blinded investigation setting, 3 raters with more than 5 years expertise in maxillofacial radiology assessed the maturation stages of 30 MRI data sets twice. Consensus reading was performed in 10 trial cases prior to the study evaluation inter- and intraexaminer reliability was estimated for all raters using Cohen’s (unweighted) kappa with a 2 week period between the different readings [34].

## Results

**Fig. 6:**
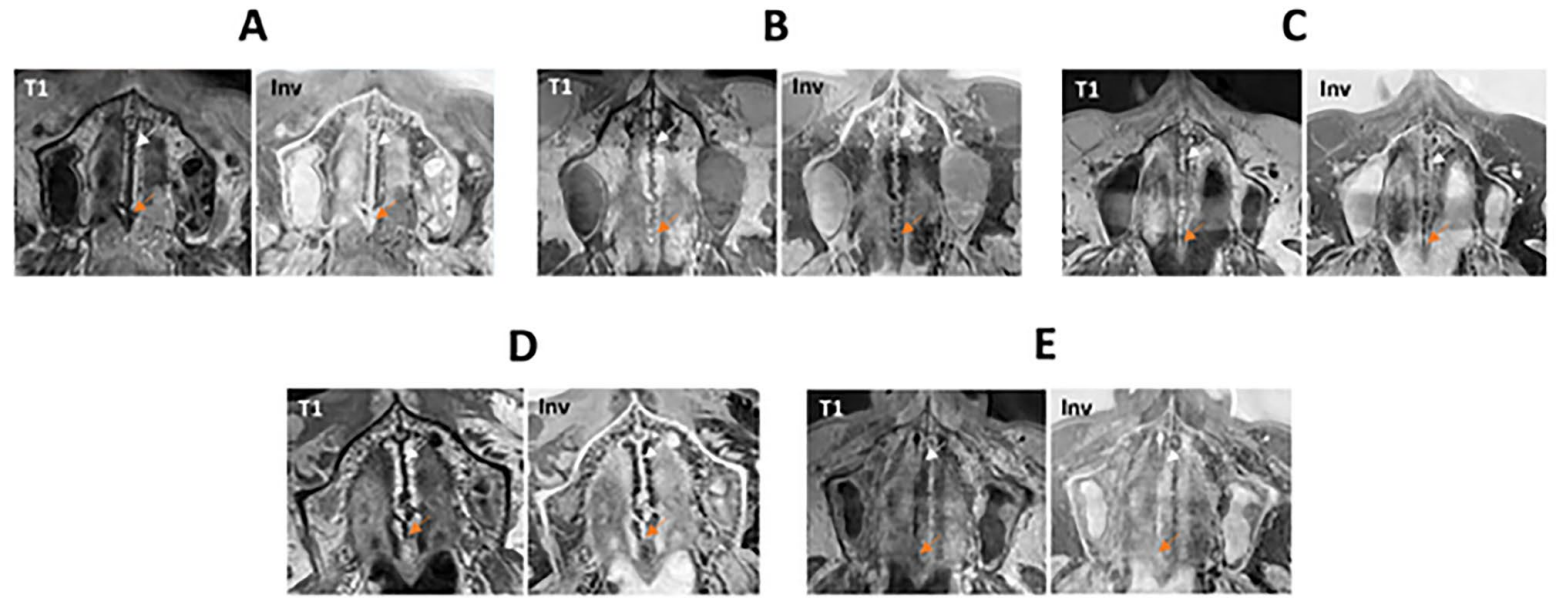
Displays the 5 different maturation stages A–E as obtained on 3 T MRI (T1 = original T1w 3 T MRI; B: inverted 3T MR image). In stages A–C, the suture is visible in both the maxillary (white arrow in A–E) and palatine bones [17](yellow arrow in A–E). **A** Stage A: the suture is straight high-density line with no or little interdigitation [17]. **B** Stage B: the suture is depicted as a scalloped high-density line with an irregular shape [17]. **C** Stage C: the suture comprised two parallel, scalloped, high-density lines close to each other [17]. **D** Stage D: sutural fusion has commenced in the palatine bone [17] (yellow arrow). **E** Stage E: the suture is totally fused and is neither visible in the maxillary (white arrow) nor palatine bones (yellow arrow) [17]

3T MRI of the midface was successfully performed in 30 patients (24–93 years; 15 females, 15 males; mean age 54 ± 17). Mean acquisition time was 07:26 min. Minor motion artefacts were only observed in a minor proportion of all patients. In patients with a very high or thick palate, we employed curved MPR reconstructions to visualise the entire palate in one axial slice as opposed to evaluating two different slices as described in the original protocol (Fig. 5). Two senior radiologists validated and critically evaluated the five patients who had received both, a high-resolution CT as the current gold standard and a 3 T MRI. Although the MR image quality does not yet match the thin-slice CT and is inferior in terms of resolution, it was deemed feasible for further analysis and interpretation (Figs. 3, 4). All 30 MRIs were rated twice by all three raters, who had been calibrated beforehand, with both, the native MRI as well as the inverted versions being presented to the raters. For the 30 MRI data sets inter-rater reliability for palatal maturation demonstrated substantial agreement, with an overall Cohens Kappa Coefficient of 0.72 [0.64; 0.80] for the first and second measurement (Table 1). Intra-rater reliability for palatal maturation equally proved substantial agreement, with weighted Kappa Coefficients being 0.69 [0.67; 0.95] (Rater 1), 0.68 [0.68; 0.95] (Rater 2) and 0.77 [0.75; 0.98] (Rater 3) (Table 1) [35]. Differential ratings were more frequent in patients with stages B, C and D while the rates were able to discriminate between stages A, and E with relative ease. By means of inversion, the MR image resembles to the suture as displayed on CBCT or conventional CT (Figs. 2, 3). The results of the inverted axial MRI slides are shown in Fig. 6.

**Table 1:** Displays the results of the intra-rater reliability and inter-rater  reliability with the 95%-CI

Intra-rater reliability	
Rater 1	0.69 [0.67; 0.95]
Rater 2	0.68 [0.68; 0.95]
Rater 3	0.77 [0.75; 0.98]
Overall	0.71 [0.76; 0.90]
Inter-rater reliability	
1 vs 2	0.66 [0.68; 0.89]
1 vs 3	0.78 [0.78; 0.95]
2 vs 3	0.73 [0.75; 0.93]
Overall	0.72 [0.64; 0.80]

## Discussion

In dental radiology, different indications, such as impacted- and supplementary teeth, orofacial clefts, pre-surgical planning among others, warrant 3D imaging [36–38]. With regard to sutural maturation status, there is no linear correlation between chronological or skeletal age and mid-palatal suture fusion [39]. Cross-sectional imaging can be beneficial to the assessment of the precise maturation stage, facilitating clinical decision making and outcome [13]. Up-to-date, axial CBCT images present the gold standard for the assessment of sutural maturation status [13, 17, 18]. As opposed to 2D panoramic images, which are routinely performed in dental medicine, CBCT creates 3D images without superimposition with other anatomic landmarks, allowing for a standardized description of mid-palatal sutures maturation [40, 41]. However, since children and young adults present a large proportion of the orthodontic patient collective, the medical indication for employing ionizing radiation has to be well-considered [38]. Children are significantly more vulnerable to long term, stochastic irradiation effects than adults, with radiation risk being inversely proportional with age [42]. With regard to radiation exposure, pronounced differences have been observed between CBCT units, with dose assessment being particularly challenging in children [43, 44]. CBCT doses in pediatric dento-maxillofacial imaging range from 103 µSv for upper and lower jaw imaging (FOV 10 × 10, small FOV) to 175 µSv for skull protocols (medium 17 × 11 and large 24 × 19 FOV) [44]. The lifetime attributable risk is particularly high in skull protocols, which have equally been reported to dramatically increase the likelihood of brain cancer in children [23, 44]. A further disadvantage seems to be that many dental CBCT units are tailored to usage in adults and many do not have imaging protocols specific for a paediatric population, resulting in elevated radiation doses for the thyroid gland, the eye lenses, the brain, and the salivary glands [42, 45]. Due to the above-mentioned disadvantages of ionising radiation in pediatric dentistry, there have been increasing efforts to implement non-ionising cross-sectional imaging into clinical routine [31]. In this pilot study we investigated the suitability of 3T MRI for the conspicuity of the hard palate and its adjacent sutures. A robust intra-, and inter-rater reliability were observed, however, our reliabilities values are not as high as those reported in other CBCT studies, evaluating sutural maturation [17, 39]. This might be attributed to the image quality of MRI, which is still inferior to CT/CBCT, particularly with regard to spatial resolution. Furthermore, dentists are not accustomed to routinely evaluate MR images, however, with respective training, the skills in evaluating dental MRI could certainly be improved. We consciously inverted the T1w MRI sequences to obtain an image impression that is comparable to CBCT, facilitating the transition from CBCT to MRI for dentists (Fig. 6). Discrepancies with regard to inter-rater reliability, were primarily found in stages B, C and D while stages A and E were scored with relative ease. This might be due to the fact that stages A (an open and wide suture, Figs. 1A, 6A) and E (a completely fused suture, Figs. 1E, 6E) are more likely a visual diagnosis with less differential diagnostic contemplations to be made. In patients with a very high or very thick palate, the respective axial slices are often not appropriate for the visualization of the entire suture in a single image. In those cases, the evaluation of at least two slices per jaw has been described by Angelieri et al. for a valid assessment of the actual anatomy [17]. We utilized curved MPR reconstructions, which are widely available on MRI scanners and in 3D post-processing tools. Those curved reconstructions and can ease the reading and increase the accuracy of the respective maturation status (Fig. 5). The fact that our approach is based on MRI data, might certainly present a limitation for many dentists, since MRI, as opposed to CBCT, is not readily available in every clinical setting. In addition, the acquisition recording time of 07:26 min is quite long, which leads to an increased risk of movement artefacts and is impractical in clinical orthodontic routine. However, with novel MRI devices and respective protocol optimization using artificial intelligence, acquisition times are likely to decrease in the near future, resulting in patient friendly examination times. Due to the retrospective design of this study the patient collective is comprised of adults only and the respective MR sequences have to be confirmed in paediatric patients to improve clinical validity. The validity of the Angelieri method in general has been questioned and alternative grading methods based on CBCT images e.g. density of the sutural area relative to soft tissues have been introduced [46]. The presented MRI protocol might, therefore, be helpful to reduce ionizing radiation in other study settings investigating the hard palate and adjacent sutures. The results of the preset pilot study display the feasibility of 3T MRI with a T1w single slab 3D TSE sequence for the depiction of the hard palate and mid-palatal suture. Inversion of the MR images yields a closer resemblance to an image impression comparable to CBCT. Further studies with larger sample sizes, including a pediatric collective, are aimed to improve and standardize non-ionizing imaging in dental radiology.
